# Implementation of Dietary Reference Intake Standards in Preschool Menus in Poland

**DOI:** 10.3390/nu10050592

**Published:** 2018-05-10

**Authors:** Joanna Myszkowska-Ryciak, Anna Harton

**Affiliations:** Department of Dietetics, Faculty of Human Nutrition and Consumer Sciences, Warsaw University of Life Sciences (WULS), 159C Nowoursynowska Str, 02-776 Warsaw, Poland; anna_harton@sggw.pl

**Keywords:** preschools, nutrition, nutrients, nutritional norms, recommendations, menu, preschool children

## Abstract

Although the nutritional value of preschool menus largely determines the proper nutrition of attending children, their nutrient composition often does not meet the standards. The purpose of the study was to assess the nutritional value of menus served in preschools throughout Poland. We analyzed a sample of 10 daily menus and inventory reports reflecting foods and beverages served in 270 full-board government-sponsored preschools. Nutrient content was calculated per child per day, and compared with 70% of dietary reference intake (DRI) for children aged 1–3 and 4–6. The content of energy, protein, fat, and carbohydrates generally exceeded 70% of DRI. The amount of vitamins was correct, with the exception of vitamin D (100% of daycare centers (DCCs) were below the recommendations); in ≤3% of preschools vitamin E, folate, and niacin were below DRI. Calcium was too low in 63% of preschools for children aged 1–3 years and in 99% for 4–6-year-olds. A shortage of iodine, iron, and potassium (especially for 4–6-year-olds) was observed in a small number of preschools. Our study highlights the need for uniform legal standards of nutrition in childcare centers, based on the current recommendations for the age group.

## 1. Introduction

Children attending full-time daycare centers (DCCs) can consume a significant proportion (50–75%) of their required dietary intake while they are in care [1]. Therefore, nutrition in DCCs has a significant effect on the proper balance of children’s diet. Studies show that the quality of nutrition in daycare facilities is not always consistent with recommendations and standards, both nationally and internationally. Nutrition analysis in 46 Australian childcare services showed that in none of them did children receive the recommended servings of vegetables, while in all institutions children were served products with high fat, sugar, and salt content [2]. Childcare centers in the USA were reported to serve inadequate amounts of whole grains, lean meat, fruit, and vegetables [3]. Analyses of the nutritional value of 300 samples of preschool meals in Serbia showed that, despite having the proper amount of energy, there was too low a share of low-energy foods (vegetables and fruits), which can promote incorrect eating habits (low consumption of fruits and vegetables) [4]. The menus in kindergartens in Granada, Spain provided the proper amount of vegetables, fruit, meat, and fish, but did not meet the recommendations for calcium and zinc [5]. Analysis of a 10-day menu from a Polish kindergarten indicated an adequate supply of energy, but too low a content of calcium, vitamin C, and iron compared to the recommendations [6].

Moreover, nutrition in childcare services also shapes the eating habits of children during a critical period of early life. Eating habits formed in childhood affect eating practices during adulthood [7]. Therefore, proper nutrition in childhood might aid in the prevention of diseases resulting from incorrect nutrition and occurring in later life [8]. One of the most common diet-related diseases in industrialized countries, as well as in Poland, is obesity caused by excessive energy consumption [9]. Compared to other European countries, Poland has a medium (14.3–17.4% in girls) to high (18.8–24.6% in boys) level of overweight and obesity in children aged 7–18 years [10]. A nationally representative study on preschool-aged children in Poland showed that obesity rates among Polish girls (7.3%) and boys (8.3%) aged 2–5 were higher than for their American (6.3% for boys) and Norwegian peers (below 5% for both, girls and boys) [11]. Secular trends of body mass index and waist circumference showed that abdominal obesity in Polish children increased significantly during the last 46 years, with a greater increase in the percentage of individuals with central obesity than with overall obesity [12]. The increasing prevalence of childhood obesity is associated with the emergence of comorbidities including type 2 diabetes mellitus, hypertension, non-alcoholic fatty liver disease, obstructive sleep apnea, and dyslipidemia [13,14,15]. This situation is alarming and requires a public health approach, if possible at an early stage. Special attention should be paid to nutrition in childcare services, with a particular emphasis on compliance with dietary norms and recommendations in the supply of energy and nutrients.

It should be emphasized that in Poland there are currently only very general legal regulations concerning nutrition-related practices for preschools regarding the minimum number of portions for dairy products (at least two servings, but the standard serving size is not fixed), meat and meat alternatives (at least one serving), fruit and vegetables (at every meal), grains (at least two servings) per day, and servings of fish per week (at least one). There are also some limitations on added sugar content in beverages prepared in DCCs from scratch (10 g per 250 mL), and on the number of fried meals per week (a maximum of two times a week) [16]. However, there is no central legal regulation concerning nutrient supply in DCCs’ menus. Existing recommendations (e.g., Polish dietary reference intake standards) are not mandatory for preschools, and employing a dietician is not obligatory. In Poland every institution is responsible for planning its own menu, so nutrition in DCCs can vary significantly. The existing data on nutrition come from analyses of individual childcare services, and have a different methodology. In some, the preschool menu is analyzed [5,6,17,18,19]; in others, children’s dietary intake [20]. Therefore, it is important to assess the quality of nutrition with a uniform methodology, and in a larger sample of DCCs located in different regions of the country. Only such an approach will allow us to assess the existing situation, find the most frequent irregularities, and formulate practical indications for improvement.

The purpose of the present study was to assess the nutritional value of menus served in preschools throughout Poland, and to compare the obtained values to the dietary reference intake standards.

## 2. Materials and Methods

### 2.1. General Information

The present study is part of an educational and research program “Eating Healthy, Growing Healthy” aimed at daycare centers, nurseries, and preschools in Poland in 2015–2017. The main goal of the project was to improve the quality of nutrition in care center facilities by increasing the knowledge and dietary awareness of personnel in participating institutions. The recruitment process included written invitations sent to childcare institutions throughout Poland (a list of institutions was obtained from dedicated government and municipal offices), as well as through a promotional campaign run on websites devoted to the education of preschool-age children. Participation in the program was free of charge for all enrolled institutions. The program offered the possibility of direct or indirect participation. Directly participating institutions were subject to supervision by a specially trained educator, who provided nutrition education for staff, analyzed nutrition-related practices in the institution before and after education, and offered written and verbal feedback. Institutions participating indirectly were provided with permanent access to the education website platform, and additionally received monthly newsletters on the nutritional issue. In total, 2638 institutions (nurseries and preschools) participated in the program, of which 1347 were directly involved. Detailed information about the “Eating Healthy, Growing Healthy” program are available in a previously published article [21].

Institutional Review Board approval was not necessary for this study as it was not deemed to be human subject research according to the University of Life Sciences Centre Institutional Review Board. No personal data were collected about children attending care facilities and institution staff; applicants (preschool directors) were informed about the purpose and scope of the research, and the possibility of withdrawing from it at any stage without giving any reason.

### 2.2. Study Setting

The present study focused on the analysis of energy and nutrient content in menus served to children in preschools (1101 institutions in total). The inclusion criteria included government-sponsored (municipal/public) institutions (778 institutions from the total) offering full board (two main meals: breakfast and lunch, and 1–2 snacks) (529 DCCs). Additional criteria for inclusion were maintaining kitchen facilities at the preschool and preparing all meals from scratch, as only in this case was it possible to receive full inventory reports covering all the food products used to prepare meals and the number of children eating on the day in the preschool. The above criteria were fulfilled by 270 preschools (25%), and data from all these institutions were analyzed in the presented study.

### 2.3. Analysis of Energy and Nutrient Content of Preschools’ Menus

The content of energy and nutrients was calculated on the basis of inventory reports and menus from 10 consecutive days (two weeks) obtained from enrolled preschools during the first visit (at baseline, before education training). In Poland, it is recommended to plan menus for mass catering (care institutions, hospitals, etc.) for a longer period (usually seven or 10 days), which ensures a better balance in nutrient content and provides suitable diversity (not repeating the same meals during this period). Therefore, the most recent two weeks’ menus (10 days) were analyzed [22]. Inventory reports included all products (description/name of the food product and its quantity in kilograms/liters/pieces) used in the kitchen to prepare all meals for children, along with the number of children who consumed these meals that day. An example of an inventory report and menu is available in the Supplementary Materials (Figure S1, in Polish/English). Based on these data, it was possible to calculate the amount of food products and their nutritional value per child per day. In our study, we calculated the nutritional value of a daily menu per child for each of 10 consecutive days, and then averaged the energy and nutrient content over one day of care per child. All calculations were conducted in the computer program Energia v 4.1 (Copyright 1997/2006 by Andrzej Miegoć, Warsaw, Poland) with the Polish Nutrition Database [23]. In total, we reviewed and analyzed a sample of 2700 documents reflecting food and beverages served to children attending 270 preschools throughout Poland. We computed the energy, macronutrients (total fat, saturated fatty acids, monounsaturated fatty acids, polyunsaturated fatty acids, cholesterol, total protein, animal/vegetable origin protein, total carbohydrates, sucrose, lactose, starch and dietary fiber), vitamins (A, retinol, beta-carotene, B_1_, B_2_, B_6_, B_12_, C, D, E, folate, niacin), and minerals (calcium, copper, iodine, iron, magnesium, phosphorus, potassium, sodium and zinc). According to national recommendations, in planning diets for groups (e.g., for children attending preschools), the reference should be the recommended daily allowance (RDA) or adequate intake (AI), and for energy-estimated energy requirements (EER) [24] these standards were used in the present study to assess the adequacy of menus.

The content of nutrients referred to Polish dietary reference intake (DRI) [24]: (1) EER for energy (kcal); (2) RDA for protein (g), carbohydrate (g), calcium (mg), copper (mg), iron (mg), magnesium (mg), phosphorus (mg), zinc (mg), vitamin A (μg, as retinol activity equivalents), vitamin B_1_ (mg), vitamin B_2_ (mg), vitamin B_6_ (mg), vitamin B_12_ (μg), vitamin C (mg), vitamin E (mg), folate (μg, as dietary folate equivalents), niacin (mg); (3) AI for sodium (mg), potassium (mg), iodine (μg), vitamin D (μg) [25], and dietary fiber (g); (4) the acceptable macronutrient distribution range for protein, fat, and carbohydrates (as a percent of total kilocalories/energy). The general population recommendations for Polish population were adopted for cholesterol and saturated fatty acids level. Polish dietary reference intakes for energy and nutrients are formulated separately for children aged 1–3 and 4–6. Children from three to six years old are enrolled in kindergarten, and the same preschool menu is planned for all attending children. Therefore, both age categories are included in the analysis of the adequacy of menus.

Accordingly to the Polish recommendation, the preschool meals should provide at least 70% of the daily energy and nutrients requirements of children, if they spend up to 10 hours there a day and receive at least three meals, including two main meals [26]. As all preschools included in this study fulfilled this criterion, the average nutrient content of the menus was compared with 70% of the dietary reference intake standards for children for each nutrient within two age categories. The percentage of kindergartens with a content of nutrients below that level was calculated. Additionally, average nutrient content was also calculated as a percent of the DRI by age [24,25].

### 2.4. Statistical Analysis

Statistical data processing was performed using Statistica version 12 (CopyrightStatSoft, Inc., 1984–2014, StatSoft Polska Sp. z o.o. manufacturers, Cracow, Poland). The Shapiro–Wilk statistical test for testing the normality of quantitative variables was used with the level of significance at *p* < 0.05. For the analyzed nutrients, the distribution measures were calculated: the lower quartile (Q_1_), the median (mean and standard deviation (SD) for data with normal distribution), and the upper quartile (Q_3_).

## 3. Results

### 3.1. General Characteristics of Preschools

The study included 270 full-board government-sponsored (public) preschools in Poland attended by 34,188 children aged 3–6 years old. All the examined preschools maintained a kitchen and prepared all meals (two main meals and 1–2 snacks) offered to children from scratch in the institution. The average financial rate per child per day was 5.8 ± 1.17 PLN (Polish zloty), which is about 1.33 euros. Only in 17 (6.3%) of 270 examined preschools was a dietitian employed; in the rest of the institutions kitchen staff or a purchasing manager was responsible for menu planning, food purchasing, and meal preparation. In total, 2700 daily menus and inventory reports were examined to calculate the energy and nutrient content of meals offered to children in childcare centers.

### 3.2. Energy and Macronutrients

Table 1 presents the amounts of energy and macronutrients provided in preschool menus, the age-specific dietary reference intake standards, the comparison with dietary reference intake (DRI) standards by age, and the percentage of preschools below 70% of DRI. The energy value of menus ranged from 903 kcal/day to 1872 kcal/day. Taking into account the energy supply recommendations for the preschool menu, in the case of 4–6-year-olds, 16 kindergartens did not meet the recommendations. The minimum protein content in the DCCs menu was 28 g/day, and the maximum 97 g/day, and the larger part was protein of animal origin. However, only in two kindergartens did the share of energy from protein exceed the recommended 20%. The amount of total fat ranged from 25 g/day to 80 g/day, whereas the percentage of energy derived from this nutrient was within the range of 23% to 45%. In 76 preschools, the share of energy from fat exceeded the recommended 35%. The minimum cholesterol content of daily menu was 92 mg, and the maximum 344 mg. Only in 33 preschool menus did the amount of cholesterol exceed the recommended level. The amount of carbohydrates ranged from 102 g/day to 263 g/day, whereas the percentage of energy derived from this nutrient varied between 39% and 63%. In 78 preschools, the share of energy derived from carbohydrates was below the recommended 50%. The dietary fiber content of menus ranged between 9.5 g to 36 g, and only two preschools did not reach the recommendation for 4–6-year-olds.

### 3.3. Vitamins

The overall vitamin composition of examined menus as well as a comparison to the dietary reference standards are outlined in Table 2. Vitamin A content in menus ranged from 367.4 μg/day to 2460.1 μg/day. The recommended level was reached in every preschool. For B group vitamins, ranges were recorded (B_1_ 0.5–1.5 mg/day; B_2_ 0.6–1.8 mg/day; B_6_ 0.8–2.5 mg/day, and B_12_ 1.0–8.2 μg/day), and no preschool with supply below the recommended values was noted. The content of vitamin C in menus was between 38 mg and 273 mg, and all examined preschools were above the recommended level. Vitamin D ranged between 0.6 and 3.5 μg/day; none of the preschools achieved the recommended amount. Vitamin E content ranged from 3.4 to 12.7 mg/day, and menus from nine kindergartens were below the recommended value. The content of folate was between 113.2 and 409.5 μg/day, and in the case of 4–6-year-old children seven preschool menus did not meet the recommendations for this vitamin. Niacin ranged between 5.5 mg and 16.5 mg/day, and one preschool was below the recommendation for children aged 4–6 years.

### 3.4. Minerals

Table 3 presents the amounts of minerals provided in preschool menus, the age-specific dietary reference intake standards, a comparison with dietary reference intake standards by age, and the percentage of preschools below 70% of DRI. Calcium daily supply ranged between 160 mg and 768 mg, and 171 preschools did not comply with the recommendations for three-year-old children, whereas for 4–6-yearolds 267 institutions did not comply with the recommendations. The content of copper in menus was between 0.5 mg and 4.1 mg, and all examined preschools were above the recommended level. Iodine ranged between 12.7 μg and 658 μg; 58 preschool menus were below this level. Iron daily supply was 4.3 mg to 15.4 mg; 16 preschools did not achieve the recommended values for three-year-old children, and 168 DCCs achieve the recommended values for 4–6-year-old children. Magnesium ranged between 126.9 mg and 556.4 mg per day; all kindergartens provided the children with the recommended amounts. Phosphorus daily supply ranged between 461.5 mg and 1324.7 mg; no menus below the recommended values were observed. The content of potassium in daily menus was between 1332.5 mg and 4421.5 mg; in the case of 1–3-year-old children, 11 kindergartens did not achieve the recommended values, while for 4–6-year-old children 74 DCCs did not achieve the recommended values. Sodium content values were from 336.7 mg to 11,669.2 mg per daily menu; only one preschool did not comply with the recommendations for three-year-old children, and three for 4–6-year-olds. Zinc daily supply ranged between 3.7 mg and 11.0 mg; no menus below the recommended values were noted.

## 4. Discussion

In Poland 84.2% children in the age group of 3–5 years (more than 1140.6 thousand) were enrolled in DCCs in 2015–2016; in 2016–2017 (due to the changes in legal regulations) 80.7% of children aged 3–6 years attended various forms of preschool education [27,28]. Those children in full-time childcare spend up to 50 h/week in nonparent care, and obtain up to 75% of their daily requirements in care services [26], leaving one-fourth to one-third to be consumed away from the care center. Nutrition outside the DCCs theoretically should complement the care service menu, especially in “key” product groups (vegetables and fruit, whole grain products, lean meat and meat alternatives, dairy), and nutrients. However, observation shows that it is not always adequate: children consume more energy and less fruit, vegetables, and milk outside of childcare centers than recommended [29]. Moreover, mothers often overestimate the nutrition quality of their child’s diet. If they continue to misperceive their children’s diet as good/healthy, they will not make any attempt to balance it better [30]. With such a high proportion of children in preschool attendance, it is imperative that childcare center providers ensure meals are healthful and nutritionally adequate. Consequently, ensuring proper quality of nutrition in care and educational facilities is crucial for the proper development of children aged 3–6 in Poland.

For maintaining a healthy body mass, an adequate supply of energy is crucial [13]. Comparisons of results from different countries are difficult due to differences in dietary reference intake standards for children, the duration of staying at the care center, and various recommendations for nutrition (nutrients supply) in the institution. For example, in Serbia the recommendation is for a supply of at least 1600 kcal/day, but meals served in the examined preschools provided an average of only 979 kcal per day [4]. The average energy value of the menus at a daycare center in South Carolina, USA ranged from 764 kcal to 929 kcal per day, with recommended levels from 665 kcal for three-year-old girls to 1167 kcal for 4–5-year-old boys [31]. However, Neelon et al. [32] reported excessive energy content in menus served in government-sponsored childcare centers in Mexico. Analysis of menus from five consecutive days conducted in one of the Polish kindergartens showed that the energy requirements for children aged 4–6 was covered in 75% [33]; however, in another Polish study the average energy content of menus from five different preschools was above the recommendation [26]. In our study, we reported exceeding the recommendations for energy supply more often than shortfalls. Only in 16 kindergartens did the menu not provide enough energy for children 4–6 years old. Additionally, large differences in the energy level were observed, reaching 200%. Even considering that children do not eat everything they get on a plate, such a high energy supply can result in excessive consumption. Kling et al. [34] found that doubling the portions increased energy intake by 24%, and increasing meal energy density by 42% increased energy intake by 40% in preschool children. Thus, intervention regarding the energy level, i.e., the adequacy of the level in terms of age, and the introduction of uniform standards for childcare institutions, is essential.

In addition to the irregularities in the energy level, we also observed an incorrect share of energy from each macronutrient. In almost one-third of preschools, the energy derived from fat exceeded the recommendation of 35%, reaching 45%. The distribution of energy from fatty acids was also not correct; in the majority of kindergartens, the share of saturated fatty acids exceeded 10% of energy. Despite the fact that the share of energy from protein occasionally exceeded 20%, with the high energy value of the menus, the dietary reference intake standards (in grams/day) were exceeded 2- or 3-fold. More adequate distribution of energy from macronutrients in preschool menus has been observed by Lazarevic et al. [4] and Neelon et al. [32]. However, our results confirmed the observations of other Polish authors [26]. The average amount of sucrose (the main added sugar) was just below 10% of total energy, but nearly one out of every three preschools exceeded that level. Despite the fact that almost 30% of DCCs had a lower than recommended share of energy from carbohydrate, fiber levels were within the recommended amount (with only two exceptions). However, the high energy content observed in our study, and the incorrect proportion of macronutrients (high proportions of fat and protein) may be a risk factor for diet-related diseases in the future [35].

Vitamins (as well as minerals) do not provide energy, but their adequate supply determines many physiological processes, as well as proper growth and development. Vitamins act as co-enzymes, antioxidants, or precursors of hormones, and are involved in many biochemical and physiological processes [36]. Vitamin A plays a key role in enhancing eyesight, growth, reproduction, and blood cell formation, and also improves the immune response [37]. In all examined preschools, the supply of vitamin A was above the recommendations. Too low a supply of vitamin A is observed rather rarely; inadequate intakes of vitamin A were found in less than 1% of the population of 3058 Brazilian children aged 2–6 years old [38], and similar results were observed in a group of 350 Polish preschoolers [39]. In our study we also evaluated the supply of B group vitamins. Thiamine (vitamin B_1_) is involved in nerve membrane function; vitamin B_2_ plays a role in metabolic reactions including carbohydrate, amino acid, and lipid metabolism. Niacin (B_3_) has an important role in regulating energy metabolism, and it also supports cognitive function. Vitamin B_6_ functions as a coenzyme for enzymes involved in amino acid metabolism, and its deficiency is accompanied by impairment of both humoral and cell-mediated immunity [40]. Folate (B_9_) is an essential factor for de novo biosynthesis of purines and plays a role in cognitive function [41,42]; deficiency of folate (as well as vitamins B_6_ and B_12_) leads to an elevation of plasma homocysteine concentrations—an early risk factor for cardiovascular diseases [43]. Vitamin B_12_ plays a vital role in preventing the onset of pernicious anemia [37], hyperhomocysteinemia [44], and deterioration of cognitive function [45]. Deficiencies of both folate and vitamin B_12_ may impair brain development in children [46]. We observed no DCCs with an inadequate supply of B group vitamins (with the exception of one preschool for niacin, and seven preschools for folate, both for children aged 4–6 years old). Our results are in line with Bueno et al. [38] and Butte et al. [47]. Vitamin C stimulates collagen synthesis and reduces potentially carcinogenic free radicals to harmless non-radical species [37]. All examined kindergartens implemented the recommendations regarding the supply of vitamin C. This finding is similar to results from Bueno et al. [38], Butte et al. [47], and Kostecka [39]. Only nine DCCs (3%) failed to meet the recommended level of vitamin E. Vitamin E is the major antioxidant in the lipid environment of cellular and subcellular membranes; deficiencies are observed very rarely [37]. Bueno et al. [38] reported inadequate supply for 15.1% of 2–3-year-old children and 28.9% of 4–6-year-olds attending public preschool. The diet of Polish preschoolers was also found to be inadequate in vitamin E [39]. However, the main problem concerned the content of vitamin D in the menus; none of the DCCs reached the recommended level. Inadequate intake was reported in more than 90% of children aged 2–6 years old in Brazil [38]; Kostecka [39], in a study conducted on Polish children, reported an insufficient intake of vitamin D (5.85 μg/day). As vitamin D has pleiotropic effects, i.e., is required for normal development and mineralization of bone and for bone remodeling [37], the introduction of fortified products to preschool menus should be considered to increase the dietary intake. It is worth stressing that the Polish reference values (adequate intake 15 µg/day) for this vitamin are very difficult to reach on a daily basis, e.g., a good dietary source of it, salmon, contains about 13 µg/100 g [25]. To prevent deficiencies in Poland, it is recommended to supplement vitamin D throughout the year or only in the period of reduced sunlight (depending on the population group and sun exposure) [48].

In addition to adequate amounts of vitamins, the correct supply of essentials minerals is a necessary condition for the proper health of children. In our study the prevalence of inadequate supply was low (≤1%) for minerals: copper, magnesium, phosphorus, sodium, and zinc for both age groups. In the case of children aged three years, too low a content of iron and potassium in menus was occasionally noted (in 6% and 4% of DCCs, respectively). Zinc is required for the catalytic activity of many enzymes, plays a role in immune function, and supports normal growth and development. Zinc metabolism is related to copper; excess zinc particularly impairs copper absorption. A daily intake of these minerals is required to maintain a steady state because the body has no specialized storage system [49]. Our results are confirmed by Bueno et al. [38] and Butte et al. [47]. However, Polish studies indicate an insufficient supply of these minerals [20,33]. Magnesium and phosphorus are known for their structural roles and for maintaining cell membranes [36], and deficiencies (especially of the latter) are rather rare. Kwiecień et al. [33] in Poland and Frampton et al. [50] in the USA noted an inadequate content of magnesium in preschool menus, especially in the case of children aged 4–6 years old. On the other hand, an adequate amount of these minerals in children’s diet was reported by others [38,47]. The average supply of sodium from food, beverages, and salt exceeded the adequate intake level, both for children aged 1–3 and 4–6. Excessive intake of sodium is reported by many authors in Poland [20,33,39] and other countries [47]. Sodium is an essential nutrient required for maintenance of plasma volume, acid–base balance, transmission of nerve impulses, and normal cell function, but increased sodium intake is associated with a higher risk of hypertension [51]. In order to reduce the amount of sodium, it is necessary to limit the salting of dishes served in kindergartens, as well as the amount of salt-rich products (e.g., processed cold cuts and hard cheese). Potassium has beneficial effects on health: a high-potassium diet lowers blood pressure in individuals with both raised blood pressure and average population blood pressure [52]. In our study the majority of kindergartens implement the recommendations for children aged 1–3 years, but in the case of children 4–6 years old, 27% of institutions failed to meet the recommendations. As our results are even higher than the observations of other Polish authors [20,33,39], it seems that more attention should be paid to the supply of good sources of this nutrient in preschool diets. However, the most problematic nutrients in our study were iron, iodine, and calcium. One in five preschools did not provide children with enough iodine; over half did not provide children with enough iron (for children aged 4–6 years). Even mild iodine deficiency may negatively affect cognitive performance, especially at a young age [53]. Data indicate that Europe has still the greatest proportion of children with inadequate iodine intake (43.9%). Salt iodization is mandatory in Poland; however, attention should be paid to other rich sources of this nutrient (e.g., fish). Another Polish study [20] reported a slightly higher (27.9%) prevalence of insufficient iodine intake in preschool children. Iron plays a key role in hemoglobin formation and oxygen transport; its deficiency may lead to anemia but also to functional impairment, affecting cognitive development [54,55]. In our study, despite the high amount of total protein and animal-origin protein, more than half of DCCs did not meet the recommendations for iron for children aged 4–6 years. Our observations are in line with other studies [33,39,50]; however, some authors reported higher intake of this nutrient [20,47]. Due to the common occurrence of insufficient supply, special attention should be paid to the rich sources of iron in preschool menus. The majority of preschools failed to meet the recommendations for calcium, especially for older children. Adequate intake of calcium is crucial for proper bone development [36]; however, the inadequate intake is very often reported in children in Poland [18,20,33,39]. Interestingly, a survey of a random sample of U.S. children demonstrated adequate calcium intake, as was seen in analyses of Belgian [56] and Spanish children [57]. Our findings indicate the urgent need for intervention. Inadequate intake of calcium with excessive intake of sodium increases the risk of not only hypertension but also osteoporosis, which is aggravated by inadequate intake of vitamin D [58].

The observed shortages in the supply of important nutrients may be associated with an inadequate share of significant product groups (dairy, vegetables, whole grain products), but also too low an energy supply resulting from insufficient supply of products in the preschool menu. This requires further research to formulate practical recommendations for improvement.

Our study provides a unique insight into the nutritional quality of menus offered to children aged 3–6 years old in preschools in Poland; however, some limitations should be addressed. The survey covered only public (government-sponsored) institutions, so the results cannot be extrapolated to all types of DCCs (i.e., private ones). In Poland, there is very limited evidence on the influence of season on menu quality. A higher level of vitamin C in menus during the summer compared to winter, autumn and spring menus was described by Orkusz and Włodarczyk [17]. However, this study was performed in one preschool only. A similar study conducted in the hospital showed no differences in the content of nutrients depending on the season [59]. In our study we analyzed 2700 daily menus that were collected during different seasons in two years. We believe that the results reflect the average nutrient contents in preschool menus. However, future studies should examine the possible influence of season on the nutrient content of menus in DCCs. The present study is focused on energy and nutrient supply on an institutional level (menu analyses). Due to a large sample of preschools (270) and collected documents (2700 inventory reports and menus), we did not measure the actual energy and nutrient intake in children. However, it was observed that children consume 50–100% of what they are offered in DCCs [60]. There is no doubt that the role of the childcare institution should be to ensure optimal nutrition for attending children. Further research should include an assessment of the actual energy and nutrient intake of children in childcare services throughout Poland.

A major strength of our study is the large sample of preschools located throughout Poland (however, the sample selection was not random). Also, the homogeneity of the tested group is an advantage: all were public (government-sponsored), full-board preschools maintaining a kitchen and preparing all meals from scratch. Furthermore, daily inventory reports were used for analysis, which show the exact amount of products used to prepare meals. The menus, which are often used in other analyses, are not fully reliable because they do not always reflect the meals actually served to children [61]. Additionally, the content of energy and nutrients was calculated as an average based on 10 consecutive days, which also validates the obtained results.

## 5. Conclusions

In the face of an epidemic of obesity, a healthy and nutritionally adequate diet should be available to children in all childcare institutions. Preschool menus largely met the recommendations regarding the supply of nutrients, especially dietary fiber, vitamins, and selected minerals. However, there are some target areas for improvement, including supply of vitamin D, calcium, iron, iodine, potassium, and especially providing an age-adequate amount of energy from recommended macronutrients. Preschools should create a context where healthy food choices are promoted: this can be achieved by making a healthy diet available for all children. Our study highlights the need for uniform legal standards of nutrition in daycare centers, based on current dietary reference intake standards for population age groups. Mandatory employment of a dietician in childcare institutions would also be a good step to improve the quality of nutrition.

## Figures and Tables

**Table 1:** Energy and macronutrients provided in preschools (*n* = 270) menus per child per day and the age-specific dietary reference intake (DRI).

Nutrient	Q_1_	Median	Q_3_	DRI for Children		% of DRI for Children		% of Preschools Below 70% of DRI for Children	
				Aged 1–3 Years	Aged 4–6 Years	Aged 1–3 Years	Aged 4–6Years	Aged 1–3 Years	Aged 4–6 Years
Energy (kcal)	1100.8	1241.4	1394.9	1000	1400	126	90	0	6
Protein (g)	39.6	45.0	50.9	14	21	329	219	0	0
Protein (% of energy)	13.7	14.5	15.5	5−15 ^2^/10−20 ^3^	10−20 ^3^	-	-	0	0
Animal protein (g)	24.0	27.4	31.7	NA	NA	NA	NA	NA	NA
Vegetable protein (g)	15.3	17.3	20.1	NA	NA	NA	NA	NA	NA
Fat (g)	39.0	44.5	53.1	33–44	31–54	141–105	150-86	0	0
Fat (% of energy)	30.1	32.9	35.3	20–35	20–35	-	-	0	0
SFA (% of energy)	11.6	12.8	14.0	as low as possible	as low as possible	-	-	-	-
MUFA (% of energy)	11.5	12.8	14.3	NA	NA	NA	NA	NA	NA
PUFA (% of energy)	4.1	4.6	5.3	NA	NA	NA	NA	NA	NA
Cholesterol (mg)	146.0	165.7	190.2	<300	<300	57	57	88	88
CHO (g)	140.1	161.6	181.0	130	130	126	126	0	0
CHO (% of energy)	49.4	51.9	54.6	50–70	50–70	-	-	29	29
Sucrose (% of energy)	7.0	8.7	11.3	NA	NA	NA	NA	NA	NA
Lactose (g)	8.1	10.2 ± 3.3 ^1^	12.9	NA	NA	NA	NA	NA	NA
Starch (g)	81.5	93.1	106.7	NA	NA	NA	NA	NA	NA
Dietary fiber (g)	13.4	16.7	18.4	10	14	161	115	0	1

^1^ mean ± SD values due to normal distribution; ^2^ for children aged 0–2 years; ^3^ for children aged three years and older; SFA saturated fatty acids; MUFA monounsaturated fatty acids, PUFA polyunsaturated fatty acids; CHO carbohydrates; NA not available.

**Table 2:** Vitamins provided in preschools (*n* = 270) menus per child per day and the age-specific dietary reference intake (DRI).

Nutrient	Q_1_	Median	Q_3_	DRI for Children		% of DRI for Children		% of Preschools Below 70% of DRI for Children	
				Aged 1–3 Years	Aged 4–6 Years	Aged 1–3 Years	Aged 4–6 Years	Aged 1–3 Years	Aged 4–6 Years
Vitamin A (μg)	749.4	1010.1	1260.6	400	450	266	236	0	0
Retinol (mcg)	210.3	252.5	308.9	NA	NA	NA	NA	NA	NA
Beta-carotene (μg)	3016.9	4044.5	5498.9	NA	NA	NA	NA	NA	NA
Vitamin B_1_ (mg)	0.8	0.9	1.0	0.5	0.6	178	148	0	0
Vitamin B_2_ (mg)	0.9	1.1 ± 0.2 ^1^	1.2	0.5	0.6	220	183	0	0
Vitamin B_6_ (mg)	1.3	1.5	1.8	0.5	0.6	313	260	0	0
Vitamin B_12_ (μg)	2.2	2.6	3.2	0.9	1.2	320	240	0	0
Vitamin C (mg)	81.0	98.3	116.5	40	50	253	203	0	0
Vitamin D (μg)	1.3	1.7	2.1	15	15	11	11	100	100
Vitamin E (mg)	5.2	6.3	7.5	6	6	109	109	3	3
Folate (μg)	183.6	214.9	252.5	150	200	105	140	0	3
Niacin (mg)	8.9	10.3	11.4	6	8	173	130	0	0.4

^1^ mean ± SD values due to normal distribution; NA not available; dietary reference intake (DRI).

**Table 3:** Minerals provided in preschools (*n* = 270) menus per child per day and the age-specific dietary reference intake (DRI).

Nutrient	Q_1_	Median	Q_3_	DRI for Children		% of DRI for Children		% of Preschools Below 70% of DRI for Children	
				Aged 1–3 Years	Aged 4–6 Years	Aged 1–3 Years	Aged 4–6 Years	Aged 1–3 Years	Aged 4–6 Years
Calcium (mg)	373.5	452.8 ± 110.1 ^1^	528.5	700	1000	65	45	63	99
Copper (mg)	0.7	0.8	0.9	0.3	0.4	288	216	0	0
Iodine (μg)	69.6	101.0	308.9	90	90	121	121	21	21
Iron (mg)	5.7	6.5	7.5	7	10	96	67	6	62
Magnesium (mg)	175.3	201.0	231.9	80	130	259	159	0	0
Phosphorus (mg)	696.7	793.6	894.3	460	500	175	161	0	0
Potassium (mg)	2114.6	2443.8	2784.8	2400	3100	103	80	4	27
Sodium (mg)	1582.9	2118.2	2783.5	750	1000	310	232	0.4	1
Zinc (mg)	5.1	5.8	6.7	3	5	199	120	0	0

^1^ mean ± SD values due to normal distribution; dietary reference intake (DRI).

## Supplementary Materials

The following are available online at http://www.mdpi.com/2072-6643/10/5/592/s1: Figure S1: An example of 1 day preschool inventory report and menu (in Polish/English translation).
