# Supplementary material for: Implementation of Dietary Reference Intake Standards in Preschool Menus in Poland
Source: Nutrients. 2018 May 10;10(5):592. doi: 10.3390/nu10050592 (PMC5986472; doi:10.3390/nu10050592)
Supplement: Supplementary file 1 [file nutrients-10-00592-s001.pdf]

Preschool XX
City Poznan

Daily Inventory Report

Nr : 66 z dnia 07-04-2015

Alimentation details

Group	Breakfast	II breakfast	Lunch	**Total**
Children	45	45	45	135
Personel			1	1
**Total**	45	45	46	136

Menu

Lp.	Meal	Menu	Cost of 1 meal	Kcal / meal
**1**	Breakfast	Tea with lemon, whole grain and regular bread with butter, rabbit patte, cucumber, apple	1,68	0,00
**2**	II breakfast	Fruit tea, pudding with raspberries, orange juice	1,86	0,00
**3**	Lunch	Cabbage soup with parsley, fried egg, potatoes, carrot with green peas, sour cucumber, multi-fruit compote	2,35	0,00
		**Full board cost per person**	5,89	0,00

Products taken from the warehouse

Breakfast

Lp.	Product	Product / description	Unit	Unit cost	Quantity	Cost
**1**	chleb razowy	Whole grain bread	Item	4,40	1,00	4,40
**2**	chleb zwykły	Regular bread	Item	2,50	2,00	5,00
**3**	cukier	Sugar	KG	2,90	1,50	4,35
**4**	cytryna	Lemon	KG	5,90	0,30	1,77
**5**	herbata	Tea	Item	1,90	0,50	0,95
**6**	jabłka	Apple	KG	2,90	3,00	8,70
**7**	masło	Butter	Item	4,70	2,00	9,40
**8**	ogorek zielony	Cucumber	KG	7,00	0,50	3,50
**9**	pasztet z królika	Rabbit pate	KG	39,98	0,94	37,58
				Total per meal		**75,65**

II breakfast

Lp.	Product	Product / description	Unit	Unit cost	Quantity	Cost
**10**	budyń	Instant pudding	Item	1,25	5,00	6,25
**11**	budyń	Instant pudding	Item	1,25	11,00	13,75
**12**	herbata owocowa	Fruit tea	Item	3,50	1,00	3,50
**13**	malina mrożona	Frozen rasperry	KG	16,38	1,20	19,66
**14**	mleko	Milk	litre	2,62	8,00	20,96
**15**	sok pomarańczowy	Orange juice	Item	6,50	3,00	19,50
			Total per meal			**83,62**

Lunch

Lp.	Product	Product / description	Unit	Unit cost	Quantity	Cost
**16**	jaja	Eggs	Item	0,75	15,00	11,25
**17**	jaja	Eggs	Item	0,70	31,00	21,70
**18**	kapusta kwaszona	Sauerkraft	KG	6,00	1,00	6,00
**19**	koper	Fennel	Item	1,50	2,00	3,00
**20**	marchewka z groszkiem	Carrots with peas	KG	4,62	2,10	9,70

Lp.	Product	Product / description	Unit	Unit cost	Quantity	Cost
**21**	masło	Butter	Item	4,70	1,50	7,05
**22**	mieszanka kompotowa	Compote mixed fruit	KG	7,66	0,90	6,89
**23**	ogórek kwaszony	Sour cucumber	package	5,00	2,00	10,00
**24**	pietruszka zielona	Green parsley	Item	1,50	1,00	1,50
**25**	włoszczyzna	Mix of vegetables	KG	4,33	1,20	5,20
**26**	ziemniaki	Potatoes	KG	1,30	7,00	9,10
**27**	ziemniaki	Potatoes	KG	1,30	7,00	9,10
**28**	łopatka b/k	Boneless pork meat	KG	18,90	0,19	3,59
**29**	łopatka b/k	Boneless pork meat	KG	18,90	0,21	3,97
			Total per meal			**108,05**
			Total meal together			**267,32**

Average cost of full board 5,81

Average cost of 1 meal 1,97

Prepared ....

Approved ....

Released ....

Received ....
